# Experimental and techno-economic investigation of industrial para-xylene plant revamping to produce meta-xylene

**DOI:** 10.1038/s41598-023-39526-3

**Published:** 2023-08-02

**Authors:** MohammadReza Khosravi-Nikou, Ali Shahmoradi, Ahmad Shariati, Meysam Hajilari, Mahsa Malek-Mahmoudi, Nemat-Allah Jafari, Abdollah Sheikh-Nezhad

**Affiliations:** 1 Department of Gas Engineering, Ahwaz Faculty of Petroleum, Petroleum University of Technology, Ahwaz, Iran; 2 Shahid Tondgouyan Petrochemical Company, Mahshahr, Iran

**Keywords:** Engineering, Chemical engineering

## Abstract

In this research, the feasibility study of revamping of simulated moving bed for paraxylene separation in ELUXYL process to produce meta-xylene using industrial Ba-faujasite exchanged adsorbent by changing operating condition (temperature and pressure) were examined experimentally and theoretically. Two different mixed-xylene feed cases (with and without presence of para-xylene) were considered. Different sets of temperature and pressure were evaluated with the help of equilibrium and dynamic experiments to obtain optimum operating condition in a favor of separation of meta-xylene. Results confirm that in the presence of para-xylene in a feed, selectivity of the adsorbent could not change towards meta-xylene. But, in the absence of para in some pressure and temperature meta-xylene was more selective than ortho and ethylbenzene. Finally, by the means of statistical experimental design method the results of all experiments were compered and an optimum temperature and pressure were found. Breakthrough experiment in optimum condition showed MX/OX and MX/EB selectivity as 1.83 and 1.15, respectively. Furthermore, the design and simulation of the real industrial SMB plant was performed in Aspen Chromatography and HYSYS software to evaluate the real performance of MX separation. Simulation results showed the final purification in SMB plant can be reached to 83.91%. At the end, for the aim of promoting purity by considering extra distillation towers the purity of meta-xylene was achieved by 96%. The economical investigation showed that by considering 700$/ton for feed supplied, the process can be satisfactory from economical point of view.

## Introduction

Mixed-xylene (three isomers of di-methylbenzene and ethylbenzene) include aromatic hydrocarbons with the general formula C_8_H_10_. The three isomers of dimethylbenzene have a benzene ring, where two methyl groups are placed in ortho, meta, and para positions. The applications of these isomers include the production of terephthalic acid using oxidation of para-xylene (PX), the production of phthalic anhydride using ortho-xylene (OX), and the production of styrene and polystyrene, which are obtained as the result of the dehydrogenation of ethylbenzene (EB)^1–4^. Meta-xylene (MX) is also used as the raw material in the synthesis of isophthalic acid (IPA) monomer. Isophthalic acid (benzene 1,3-dicarboxylic acid) is an organic dicarboxylic acid that is industrially produced from the oxidation of MX. IPA is used as an additive in the preparation of polyethylene terephthalate (PET) to improve its thermophysical properties. To utilize the best method for the separation of xylene isomers, knowledge of their chemical and physical properties is necessary^4–6^. Table 1 summarizes physiochemical properties of these isomers. According to these data, it can be seen that many physiochemical properties of these isomers are the same. This has caused the separation of these isomers to be considered as one of the challenging separations in the petrochemical industry. Among the separation methods, the distillation method cannot used as benefit separation method due to the close boiling points. Although the crystallization method has been used industrially for separation of xylene isomers, it is not considered as the suitable method due to the low percentage of product recovery and the high energy demanded. Adsorption process is currently used as the best separation method for this purpose. Although these methods are mainly used for the production of para-xylene, they can also be effective in the separation of other isomers^1,2,4,7^.

**Table 1:** Xylene isomers, ethylbenzene physical properties^2,8^.

Isomers	PX	MX	OX	EB
Kinetic diameter (Å)	6.7	7.1	7.4	6.7
Boiling point (K)	411.5	412.3	417.6	409.3
Freezing point (K)	286.4	222.5	248	178.2
Dipole moment (D)	0	0.36 (liquid)	0.62 (gas)	0.59
Polarizability (cm^3^)	13.7	14.2	14.9	14.2
Density at 298 K (g cm^-3^)	0.858	0.861	0.876	0.867

Regarding the production of Meta-xylene, some methods had been introduced, such as Clathration, sulphonating, coloration and decolorating, which have not reached the industrial stage due to technical problems^6,9^. In 1968 the Japan Gas Chemical (JGC) presented the extraction method as one of the industrial methods of meta-xylene production. In this method, HF-BF3 was used as the solvent to form co-molar complex in the form of MX-HF-BF3. Although this process used to produce 99% pure meta-xylene, it was obsoleted due to the environmental pollution^10^. Finally, UOP company, as a pioneer in para-xylene production industry by means of adsorption process and simulated moving bed (SMB) technology, introduced the new SMB process with the aim of separation of the meta-xylene in 1995 namely “MX-Sorbex” process, with the minimum purity and recovery of 99.5% and 95%, respectively^11^.

In the SMB process, both the process and the adsorbent are vital parameters to determine separation efficiency. Enormous researches have been performed to study the xylene isomer separation through the adsorption, in which various type of adsorbents have been investigated. In general, zeolite type X and also in the form of cation exchanged, BaX and BaKX adsorbed para-xylene more selectively than other isomers. Some other types of zeolites like HZSM-5, Na-Beta, and Beta have same behavior in facing with xylene isomers. The SiO_2_/Al_2_O_3_ ratio in these adsorbents are varied from 1.5 to 2.5^5,7,8,12–15^. In the case of MX-selective adsorbent, Rasouli et al. utilized NaY adsorbent in separation of meta-xylene in two forms of micro and nano sized adsorbent. They concluded that NaY adsorbents are selective to MX isomer and nano sized one can improved selectivity parameters of MX towards other isomers. In the case of nano sized adsorbent with the SiO_2_/Al_2_O_3_ ratio of 4.8 at temperature and pressure of 130 °C and 8 bar (as an optimum point) the MX/PX, MX/OX, and MX/EB selectivity factors were reported 2.9, 3.16, and 6.88, respectively^16,17^. Some other researches on NaY, NaAgY, and NaY/LiY also show same behavior of MX-selective in facing with mixed-xylene components^8^. Khabzina et al.^5^ implemented comprehensive research on various ion-exchange zeolites and reported a vast laboratory library data by implementing three briquette experiments for 68 adsorbents. One of the most important parameters in adsorption and separation of xylenes by means of zeolites is the water content of the adsorbent. The presence of water molecules increases the acidic property of Bronsted acid sites due to increasing the polarity and strong electrostatic field between the alumina framework and its cation exchanged on the adsorbent. It caused to change interaction the adsorbate-adsorbent which is resulted on the adsorbent selectivity and mass transfer rate^18^. The optimum range of the water level in the adsorbent in the case of xylene separation were determined as 3 to 6 wt%. Some researchers proposed detail manner to determine and controlling humidity level of the adsorbent during experiment procedure^1–3,15,19–22^.

Much research has been investigated on the SMB process from modeling, simulation, and optimization points of view. Minceva et al. utilized both an equivalent true moving bed (TMB) approach and simulated moving bed (SMB) approaches to demonstrated adsorptive separation process of the SMB technology. They concluded that TMB approach can visualize the SMB process in the faster and easier way^4,23–25^. In the case of design SMB process, triangle theory and separation volume methods were introduced as appropriate methods in case of no mass transfer resistance and existing mass transfer resistance, respectively^26–28^. Moreover, two-level steady-state optimization algorithm implementing SQP algorithm was proposed by Shen et al. for achieving minimum desorbent consumption and maximum productivity^29^.

Ahmadipour et al. investigated adsorption behavior of mixed-xylene and para-diethylbenzene on industrial SPX-3000 adsorbent which is utilized in ELUXYL process of separation of para-xylene. They studied the effect of temperature on the adsorption behavior of mixed-xylene in liquid phase in both thermodynamic and kinetic points of view. They found that at 25 °C, PX is the fastest component that absorbed the highest loading capacity (71.65 mg/g). Langmuir and pseudo-second-order models show the better fitting for interpreting experimental data in equilibrium and kinetic mode, respectively^3^. In same result with Tournier et al.^15^, they confirmed that by changing operating conditions selectivity factor values can slightly be different. In another study by Shahmoradi et al. modeling, simulation and optimization of the industrially separation of the ELUXYL technology were performed by the help of the results of experiments at real operating conditions (175 °C and 9 bar). Langmuir multi-component isotherm model were determined to describe the adsorbent equilibrium data under operating conditions. Also, detail model for determining mass transfer resistance were proposed in their model^2^. Finally, they performed hierarchical method comprised of both thermodynamic and kinetic evaluation to determine the applicability of the adsorbent in industrial scope by the help of xylene separation using SPX3000 adsorbent at optimum condition. Both batch and dynamic setups and procedures were determined to achieve better insight about adsorption process. Furthermore, the water level of the adsorbent was determined as 4 wt% in the case of this special adsorbent^1^.

The aim of this work is to investigate the possibility of using the presence plant for the separation of paraxylene in order to produce meta-xylene by changing the operating pressure and temperature conditions as a case study. For this purpose, industrial Ba-exchanged faujasite adsorbent of ELUXYL (namely SPX-3000) adsorbent, which is utilized for separating para-xylene, were used to study the changing its selectivity toward meta-xylene by means of changing the operating conditions in two different feed cases. Also, using the configuration data of an existing industrial separation unit related to the ELUXYL process, the results of the experiments were simulated and optimized to find the actual results and efficiency of the proposed method on the industrial scale. Furthermore, the possibility of coupling of distillation method with the SMB was investigated. Finally, techno-economic evaluation was presented to the proposed methods.

## Materials and methods

### Materials

Meta-xylene, para-xylene, ortho-xylene, and ethylbenzene chemicals were purchased from Merck company with the purity of minimum 99%. These chemicals are used as received without further purification. Iso-octane was purveyed from Samchun company with the minimum purity of 98%. This material was used as the solvent. Para-diethylbenzene (PDEB) was supplied from Iranian Petrochemical Complex. In the case of the adsorbent, industrial type of Ba-faujasite exchanged namely SPX-3000 with the average particle size of 0.47 mm were utilized.

### Experimental section

For the study of the separation of the meta-xylene with the help of para-xylene production adsorbent, two types of experimental case were considered. Case1 study the feasibility of separation of the meta-xylene from mixed-xylene feed and the other case (Case2) was investigated the separation of meta-xylene from raffinate (which did not include para-xylene) feed. For this purpose, both type of batch and dynamic adsorption experiments were implemented. In all experiments, the effect of pressure and temperature on the adsorption capacity and selectivity of the MX separation were investigated. Based on the previous studies on this special industrial type adsorbent, 4 wt% of adsorbent humidity was determined as the optimum value.

#### Batch and dynamic set-ups

Equilibrium behavior of the adsorption is determined from batch experiment method. Multi-component single-point batch adsorption experiments were considered to obtain the adsorption capacities of each isomer at each temperature and pressure point. For the aim of investigation of dynamic adsorption behavior, breakthrough set-up was utilized. Selectivity was concluded from breakthrough experiments. The scheme of the batch and dynamic set-ups, procedure of experiments, and method of analysis can be found in *Supplementary Information.* Also, same procedure for calculating selectivity factor from breakthrough curves, which are reported by other researchers^1,30^, were used.

In order to investigate the pressure and temperature effects, the following procedure was considered. The adsorption behavior in the 5 different pressure points (3, 5, 7, 9, and 12 bar) and ambient temperature were studied in the first step. After the determination of the adsorption capacities of each isomer and selectivity factors from the first step, with the help of design of experiment (DOE) method, the three optimum pressure in the case of the most efficient adsorption capacity and selectivity points in favor of the meta-xylene were selected to study the effect of the temperature. Then, three different temperature points were examined in each desired pressure point to form 9 more different points. After obtaining optimum point in a favor of meta-xylene separation, isotherm of each isomer is calculated through batch experiment.

For the case1, real industrial composition of the mixed-xylene feed was prepared. 50% MX, 20% OX, 20% PX, and 10% EB were mixed to prepare mixed-xylene feed (compositions are based wight percent). Case2 uses real raffinate composition as a feed, which contains 62% MX, 25% OX, and 13% EB (compositions are based wight percent). Each solution was diluted in iso-octane to prepare 20 wt% concentrated solution. In the case of single-component isotherm experiments, each isomer was diluted in iso-octane in the concentration range of 0.5–8 wt%. For each batch experiment, the ratio of the solution to adsorbent was considered as 4:1 in weight percent. In the case of breakthrough analysis feed flowrate fixed at F = 20 cm^3^min^–1^ (${}_{-}^{+} 0.2{\%}$), and the case1 feed composition was comprised from 20.07% PX, 45.29% MX, 20.51% OX, 10.10% EB, and 4.03% i-octane (compositions are based volume percent). For the case2, 56.1% MX, 23.67% OX, 14.3% EB, and 5.93% i-octane were considered (compositions are based volume percent).

### Design and simulation procedure

**Figure 1:**
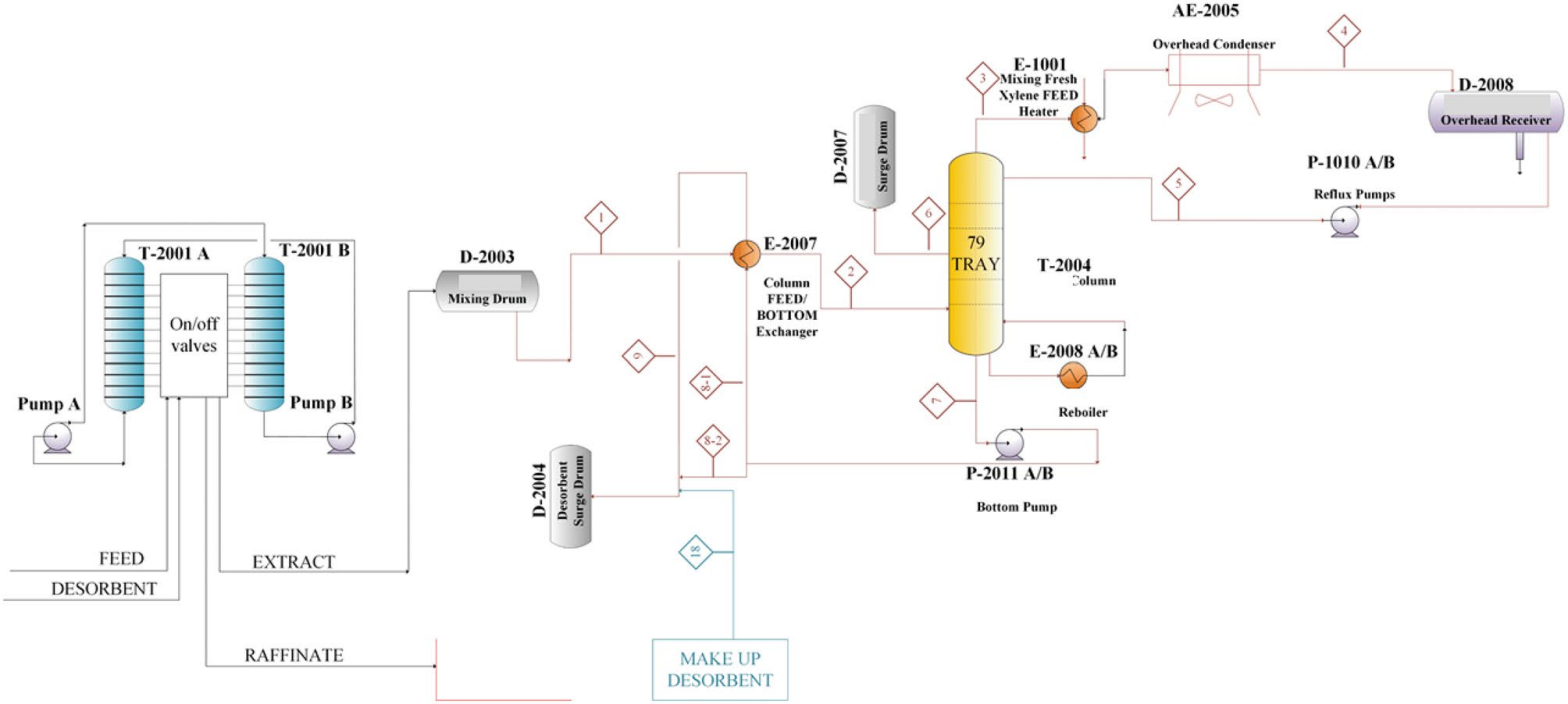
scheme of the industrial-scale SMB plant.

In this research, one of the existing ELUXYL petrochemical plant was utilized to study the performance of separation of meta-xylene at the calculated optimum operational condition. Figure 1 shows the schematic of the plant. In this research, an existing industrial para-xylene separation plant was utilized in term of study the real behavior of the process if its uses change to separation of meta-xylene. This unit is designed based on ELUXYL process and the configuration of the plant is shown in Table 2. The schematic of the SMB columns and downstream are shown in Fig. 1. SMB includes two towers which contains 12 beds. These two towers are connected to each other with a line from the bottom of one to the top of other to form close loop circulation. Each raffinate and extract streams are sent to the distillation towers to separate desorbent component from xylene isomers.

**Table 2:** Configurational parameters of the industrial-scale PX production Eluxyl SMB unit.

Parameters	Value
Length of each column (L_c_)	130 (cm)
Diameter of each column (d_c_)	480 (cm)
Density of adsorbent (ρ_p_)	860 (kg/m^3^)
Bed porosity ($\upvarepsilon$_b_)	29%
Macro porosity ($\upvarepsilon$_M_)	28.2%
Micro porosity ($\upvarepsilon$_m_)	15%
Average diameter of particle (d_p_)	0.65 (mm)

In case of SMB process design, two stand wave design and triangle theory methods are developed. Triangle theory are divided into two separation region and separation volume methods. In separation region method, which is utilized for the cases that consist with equilibrium theory, the flowrate of two zones (zone 1 and 4) and switching time are fixed and optimum performances are determined by computing flowrates of zone 2 and 3. In the case of existing mass transfer limitation, separation region method can only propose initial guess. Separation volume method calculates real optimum condition by determination of optimum values of zone 1 and 4 flowrates^4,31^.

In term of changing the unit application to separate meta-xylene at optimum operating point which is conducted from experimental step, it is necessary to determine new operational condition (flowrates and switching time). At the first step, separation region method was used to obtain initial guess of the operational condition through ASPEN Chromatography software. After that, optimization procedure was carried out to determine optimum flowrates of inlet and outlet streams. Detail information about mathematical model, simulation, and design procedure of the SMB process can be found elsewhere^2^. Also, simulation of the distillation towers in downstream of the SMB process are carried out by the HYSYS software.

## Results and discussion

### Case 1 experimental results

Equilibrium adsorption capacities and selectivity factors at different pressure and room temperature are shown in Table 3. According to these data it can be concluded that pressure cannot change the selectivity of the adsorbent toward MX isomer. By considering these results, three different temperature points are selected to study the effect of the temperature conjugated with pressure by means of DOE method. The 100 to 200 °C temperature interval was considered to remain feed as liquid. Three different temperatures as 100, 150, and 200 °C and three different pressures as 8, 10, and 12 bar are selected to comprise 9 selected points. Adsorption capacities and selectivity factors of these selected points are demonstrated in Table 4. As can be seen, it may be concluded that in the presence of the para-xylene isomer in the feed, there is no chances for separation of meta-xylene. Also, this fact can be proved that in the case of para-xylene separation, the main reason is the type of exchanged cation Ba^2+^ of the adsorbent. This cation can change the affinity of the adsorbent toward the para-xylene in the competitive adsorption. The results are confirmed that by the means of Ba-exchanged zeolite adsorbent, meta-xylene cannot be selective in comparison to para-xylene. So, this case cannot be applicable.

**Table 3:** Pressure effect on adsorption capacities and selectivity factor.

Operating conditions		Case1							Case2				
Pressure bar	Temperature ($^\circ$C)	${q}_{PX} (mg/g)$	${q}_{MX} (mg/g)$	${q}_{OX} (mg/g)$	${q}_{EB} (mg/g)$	PX/MX	PX/OX	PX/EB	${q}_{MX} (mg/g)$	${q}_{OX} (mg/g)$	${q}_{EB} (mg/g)$	MX/OX	MX/EB
3	25	11.638	17.766	9.312	5.24	1.372	1.213	0.805	23.864	11.5	7.8	0.7683	0.5180
5	25	10.486	13.958	11.722	8.464	1.579	0.849	0.422	21.84	8.77	6.988	0.9322	0.5321
7	25	10.3	12.264	5.836	4.464	2.156	1.804	0.971	1.812	0.9	4.552	0.7478	0.0660
9	25	10.736	15.568	9.264	5.396	1.505	1.123	0.666	19.244	6.178	6.354	1.1619	0.5047
12	25	8.1	9.052	5.124	4.636	2.282	1.600	0.722	22.72	5.872	5.612	1.4563	0.6787

**Table 4:** Pressure and temperature effect on adsorption capacities and selectivity factor.

Operating conditions		Case1								Case2				
Pressure bar	Temperature ($^\circ$C)	${q}_{PX} (mg/g)$	${q}_{MX} (mg/g)$	${q}_{OX} (mg/g)$	${q}_{EB} (mg/g)$	PX/MX	PX/OX	PX/EB	Temperature ($^\circ$C)	${q}_{MX} (mg/g)$	${q}_{OX} (mg/g)$	${q}_{EB} (mg/g)$	MX/OX	MX/EB
8	100	23.172	0.676	0.404	0.528	1.009	1.61	0.709	75	75.34	35.2	20.17	1.1218	1.1065
8	150	39.384	24.592	20.24	12.78	1.5	2.9	1.182	137.5	59.78	19.09	10.69	1.4624	1.0246
8	200	54.832	10.34	11.696	11.504	1.106	1.313	0.603	200	57.83	17.39	11.30	1.3431	1.0361
10	100	35.9	1.078	0.82	17.264	1.168	1.46	1.094	75	74.05	22.49	15.488	1.1824	1.1042
10	150	38.608	4.16	4.318	5.708	1.32	1.67	1.3	137.5	35.42	11.048	9.06	1.3849	0.8866
10	200	17.996	6.766	0.124	1.87	1.184	1.607	0.833	200	63.50	19.126	12.88	1.2657	1.1016
12	100	17.244	4.588	9.568	7.528	1.076	1.08	0.61	75	62.34	18.71	13.88	0.5726	0.9762
12	150	28.42	7.536	4.878	3.984	1.372	0.976	0.58	137.5	29.82	8.10	7.59	0.2683	0.7451
12	200	30.176	12.252	6.308	4.48	1.311	1.297	0.738	200	52.50	21.41	12.69	0.4649	0.9865

### Case 2 experimental results

The pressure effect on raffinate feed and room temperature are summarized in Table 3. The results show the probability of the chance in a favor of meta-xylene separation in high pressure condition. The temperature effect was investigated in same as previous manner, too. The temperature interval of 75 to 200 $^\circ$C was considered and 9 selected pressure and temperature points were determined through statistical DOE method. 8, 10, and 12 bar were chosen for pressure points and 75, 137.5, and 200 $^\circ$C were determined for the temperature. Adsorption capacities and selectivity factors are concluded from batch and dynamic experiments are presented in Table 4. Considering both temperature and pressure effects are demonstrated that in the range of 8 to 10 bar and 75 to 200 $^\circ$C meta-xylene can be adsorbed on adsorbent selectively. Adsorption loadings are shown appropriate capacity of the adsorbent in face of raffinate components. Although the selectivity factors of MX to OX and EB is less than real separation of para-xylene application case, these values can help to purify meta-xylene more than its concentration in raffinate stream. According to experimental results, mathematical optimization was performed to find the optimum temperature and pressure point.

**Figure 2:**
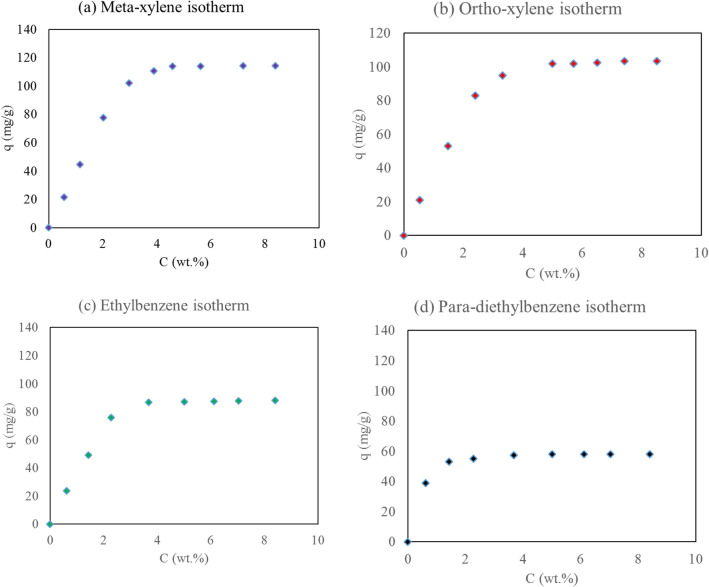
Isotherm results of (**a**) MX, (**b**) OX, (**c**) EB, (**d**) PDEB components at 185 $^\circ$C and 8.7 bar.

**Figure 3:**
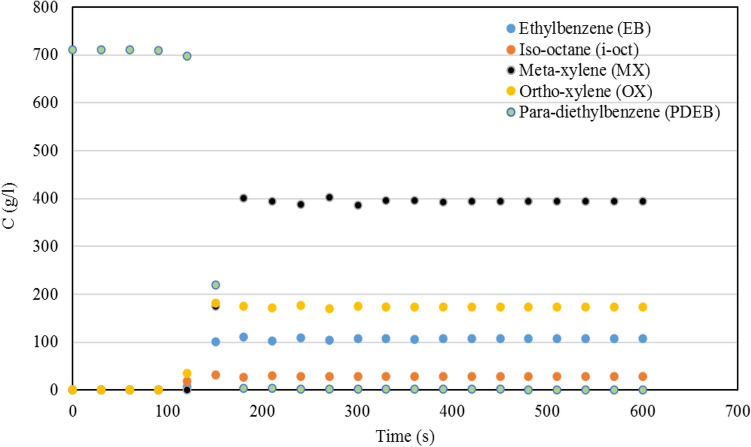
Breakthrough curve results at 185 $^\circ$C and 8.7 bar.

Response surface method (RSM) is a statistical method to define experimental design based on mathematic model. This method investigates the interaction between main factors and estimate the effect of these interactions. This method can be utilized in the case of optimization, fault detection, and etc. Central cubic design (CCD) is sub-method of RSM which consider more reliable surface response inside and outside of the main interval. By the means of CCD method, pressure and temperature were considered as factors and the adsorption of EB, OX, and MX were specified as three surfaces (table S-2). Detail of experimental design are illustrated in *Supplementary Information*. Quadratic model was determined as the benefit model to describe experimental data and also, analysis of variance (ANOVA) of the surfaces are confirmed the concluded result (tables S-6, S-8, and S-10). By considering the quadratic model for description of the data, an optimization for the aim of minimizing EB and OX adsorption and maximizing MX adsorption were carried out. The optimum pressure and temperature which is conducted from experimental design method are studied experimentally. Results of model and experiment of the adsorption data and mass transfer analysis are shown in Table 5. So, these pressure and temperature were chosen as optimum condition due to verification of model results by the experimental data. Furthermore, isotherm of each isomer was determined experimentally through batch analysis method and breakthrough curve was conducted experimentally from dynamic analysis method which are shown in Figs. 2a–d and 3, respectively.

**Table 5:** Results of RSM analysis and experimental data of single point adsorption and isotherm at optimum condition and kinetic model data.

Results of RSM and Experiment Validation						
Number	Temperature ($^\circ$C)	Pressure (bar)	EB	MX	OX	
Design expert prediction	185.3	8.77	11.06	58.62	16.44	
Experimental measurement	185	8.7	11.304	57.836	17.392	
Error %	± 1%	± 5%	± 2.2%	± 1.34%	± 5.79%	

Thermodynamic and Kinetic Parameters						
Components	Langmuir parameters		Mass transfer analysis			
	$K (\frac{{m}^{3}}{mol})$	${q}_{m} (\frac{kg}{kg})$	${k}_{ext}(\frac{1}{min})$	${k}_{int}(\frac{1}{min})$	Biot number	$Pe$
MX	4.7786	0.1451	432.9	1.8	240.5	1500
OX	1.6585	0.1451	435.708	1.82	239.4	1500
EB	2.6477	0.1451	439.39	1.84	238.8	1500
PDEB	6.1	0.1199	384.67	1.83	210.2	1500

### Design and simulation results

As mentioned before, due to the existence of the tower and downstream equipment, the design of the plant limited to the determination of the operational conditions (flowrates and switching time). For the aim of design and simulation, the loading of each isomer shall be determined mathematically. Multi-component Langmuir isotherm model (Eq. 1) can describe the loadings conducted by equilibrium experiments, reasonably.

1 $${q}_{e.i}= \frac{{q}_{m.i }{K}_{L.i}{C}_{L.i}}{1+ \sum_{j=1}^{N}{(K}_{L.j }{C}_{L.j)}}$$

where, ${q}_{m}$ is the saturation adsorption capacity *(mg. g*^*-1*^*),* ${C}_{L.i}$ is the equilibrium concentration (mg/g), ${K}_{L.i}$ is Langmuir constant (g/g),${q}_{e.i}$ is monolayer adsorption capacity for component (mg/g), *N* is total number of components in the solution^32^. This equation specified adsorption capacity and affinity parameter of each isomer in face of the adsorbent. These parameters are presented in Table 5, also, related ARD% of each isomer are shown, too. Another important parameter for modeling and simulation of SMB process is mass transfer resistance. This parameter is divided into two internal and external diffusion mechanism. Diffusing components from bulk of the fluid into the stationary film around the adsorbent particle is classified as external mass transfer rate. Due to the different types of the adsorbent particles, two meso and micro porosities can be formed^1^. In all cases diffusing adsorbates from meso to micro pores are considered by equilibrium theory. So, internal mass transfer resistance is reduced to diffusing components from stationary film around particles into mesopores of the adsorbent. Mass transfer resistance can be obtain based on procedure proposed by Shahmoradi et al.^2^. Results for the mass transfer rate are presented in Table 5. As can be seen, due to the higher value of the Biot number external mass transfer can be ignored in this system.

#### Design of SMB process

**Figure 4:**
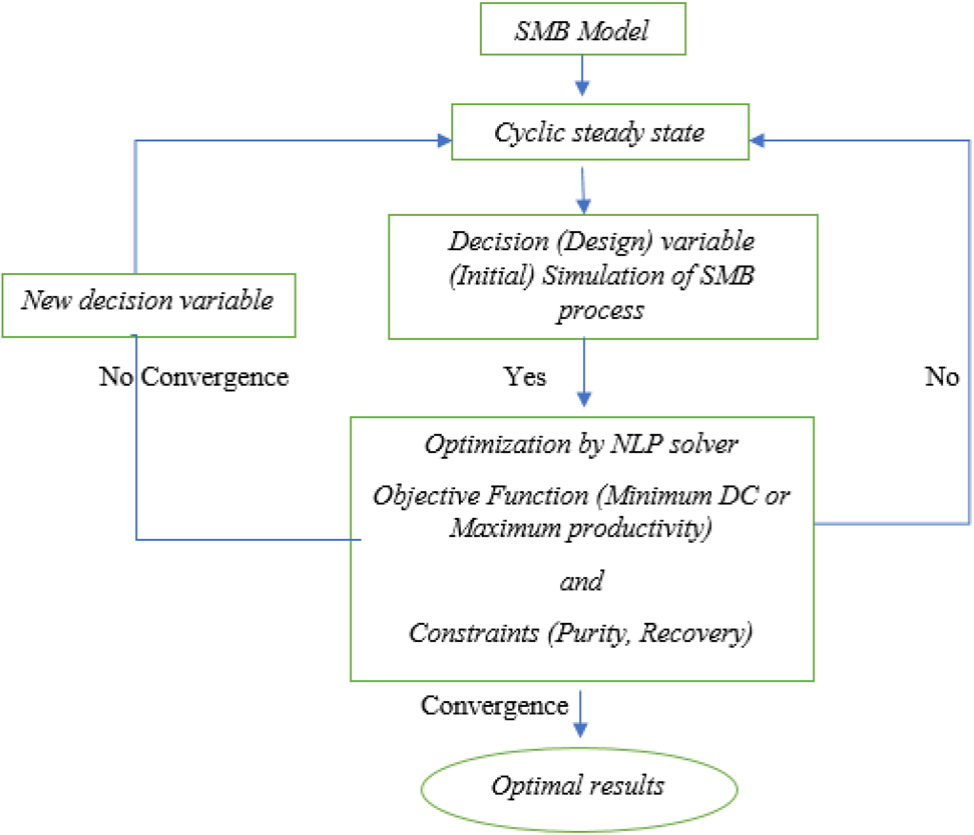
Dynamic framework optimization for obtaining optimum operational conditions.

**Figure 5:**
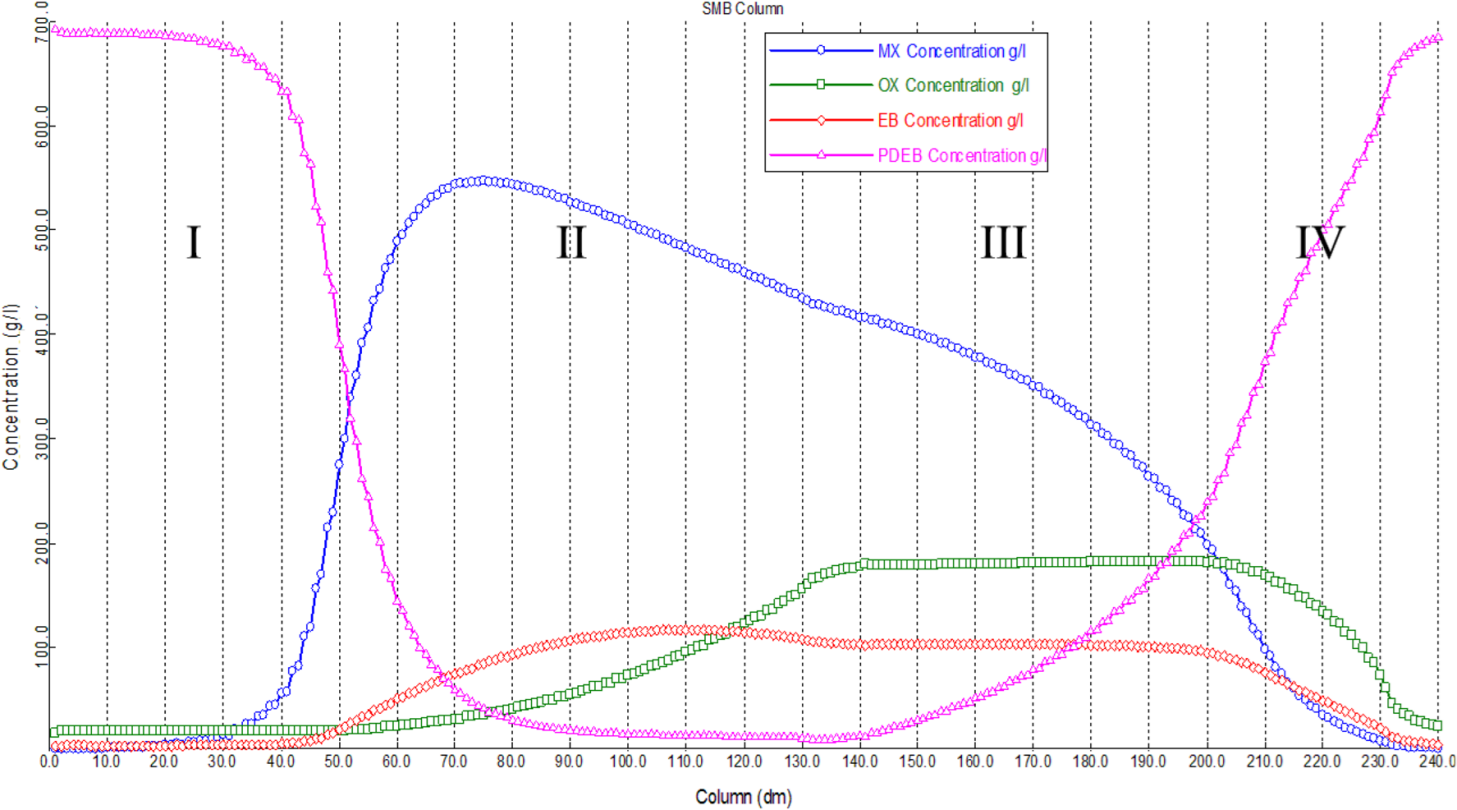
Concentration profiles at the end of switching time interval under CSS condition at optimum operational condition.

Data concluded from experiments and configuration data of the SMB towers were implemented in Aspen Chromatography software. Initial guess of the operational conditions was obtained by solving equivalent true moving bed model, which resulted triangle shown in Fig. S-3. To obtain optimum operational condition based on separation volume, an optimization process was implemented through Aspen Chromatography. Desorbent, extract and recycle flowrate are considered as design variables. Purity and recovery of meta-xylene in extract section were considered as constrains, and finally the minimum desorbent consumption was selected as the objective function (Fig. 4). Minimizing desorbent consumption is proposed favorable result than maximizing productivity in this dynamic optimization. First, the constrains were considered as 99% and 96% as meta-xylene purity and recovery, respectively. Due to un-convergence of the dynamic optimization, constrains were changed until the optimization converged meet 80% and 70% for meta-xylene purity and recovery constrains, respectively. Initial values of flowrates concluded from triangle theory, boundary values and final results of optimization are shown in Table 6. Concentration profile at the end of the switching time (Fig. 5.) demonstrated that meta-xylene profile is descended from zone II to zone IV. Also, ortho-xylene and ethylbenzene profiles are increased from zone II to zone IV. This existence of all isomers in zone II and zone III is resulted from low selectivity parameter of MX to OX and EB which is pollute extract stream and reduce purity and recovery factor of meta-xylene. Results of simulation of the downstream the SMB are summarized in Table 7. In the downstream of the SMB tower (Fig. 1), the extract stream enters the distillation tower containing 79-tray to purify from para-diethylbenzene. Purified meta-xylene rich stream is drawn from tray number 76 (stream No. 6, which is shown in Fig. 1), and para-diethylbenzene is separated at the bottom of the tower (stream No. 7, which is shown in Fig. 1). As can be seen from Table 7, the flowrates, temperature and pressure of the all streams and distillation tower are approximately the same as real condition of the unit. This is due to the minimizing change in the utility and maintenance of the unit and finally economical points of view.

**Table 6:** Results of optimization for determination of optimum operationing condition.

Decision Variable	Initial Value	Lower Bound	Upper Bound	Min. DC
*Q*_*F*_ $(\frac{{m}^{3}}{h})$	175.33	–	–	175.33
*Q*_*S*_ $(\frac{{m}^{3}}{h})$	364.512	300	400	350.497
*Q*_*EX*_ $(\frac{{m}^{3}}{h})$	349.512	350	400	366
*Q*_*Recycle*_ $(\frac{{m}^{3}}{h})$	815.478	785	844	804.27
Performance Parameters				
*Purity of PX (%)*	69.15			83.91
*Recovery of PX (%)*	60			70.24
*DC (kg /kg)*	11.874			10.048
*Productivity* $(\frac{Kg}{{{m}^{3}}_{ads}hr})$	226.185			267.45

**Table 7:** Data of downstream of the SMB tower conducted from simulation and plant data.

Stream No		1	2	3	4	5	6	7	8–1	8–2	9
Real plant data (for separation of PX)											
Temperature	^O^C	175	194	160	70	70	152	222	221.9	221.9	193.3
Pressure	bar	5.5	5.1	1.75	1.67	7.6	1.79	2.35	5.3	5.3	4.6
Flowrate	kg/h	301,200	301,200	214,500	214,500	215,000	116,317	184,873	182,276	2597	182,276
Simulation results											
Temperature	^O^C	175	194	161	70	70.25	162.9	211.8	212.1	212.1	193.9
Pressure	bar	5.5	5.1	1.763	1.683	7.613	1.832	2.363	5.3	5.3	4.6
Flowrate	kg/h	301,200	301,200	214,400	214,400	214,600	116,400	184,900	144,700	40,200	144,700
MX	wt%	37.67	42.47	83.92	83.92	83.95	83.91	10.63	10.63	10.63	10.63
OX	wt%	5.15	5.81	4.46	4.46	4.47	8.26	3.92	3.92	3.92	3.92
EB	wt%	3.17	3.58	11.62	11.62	11.59	7.83	0.27	0.27	0.27	0.27
PDEB	wt%	54.00	48.15	0	0	0	0	85.18	85.18	85.18	85.18

Finally, meta-xylene with the purity of 83.91% can be obtained from existing unit and by changing operating condition of the SMB tower. It is obvious that this purity if not acceptable and this process is not qualified. But it can be considered as pre-treatment stage to achieve meta-xylene with higher purity due to the promoting MX raffinate feed purity from 59.22% to 83.91%. In this study, two more distillation stage are designed to obtain higher purity of the meta-xylene goal.

#### Design of distillation tower

**Figure 6:**
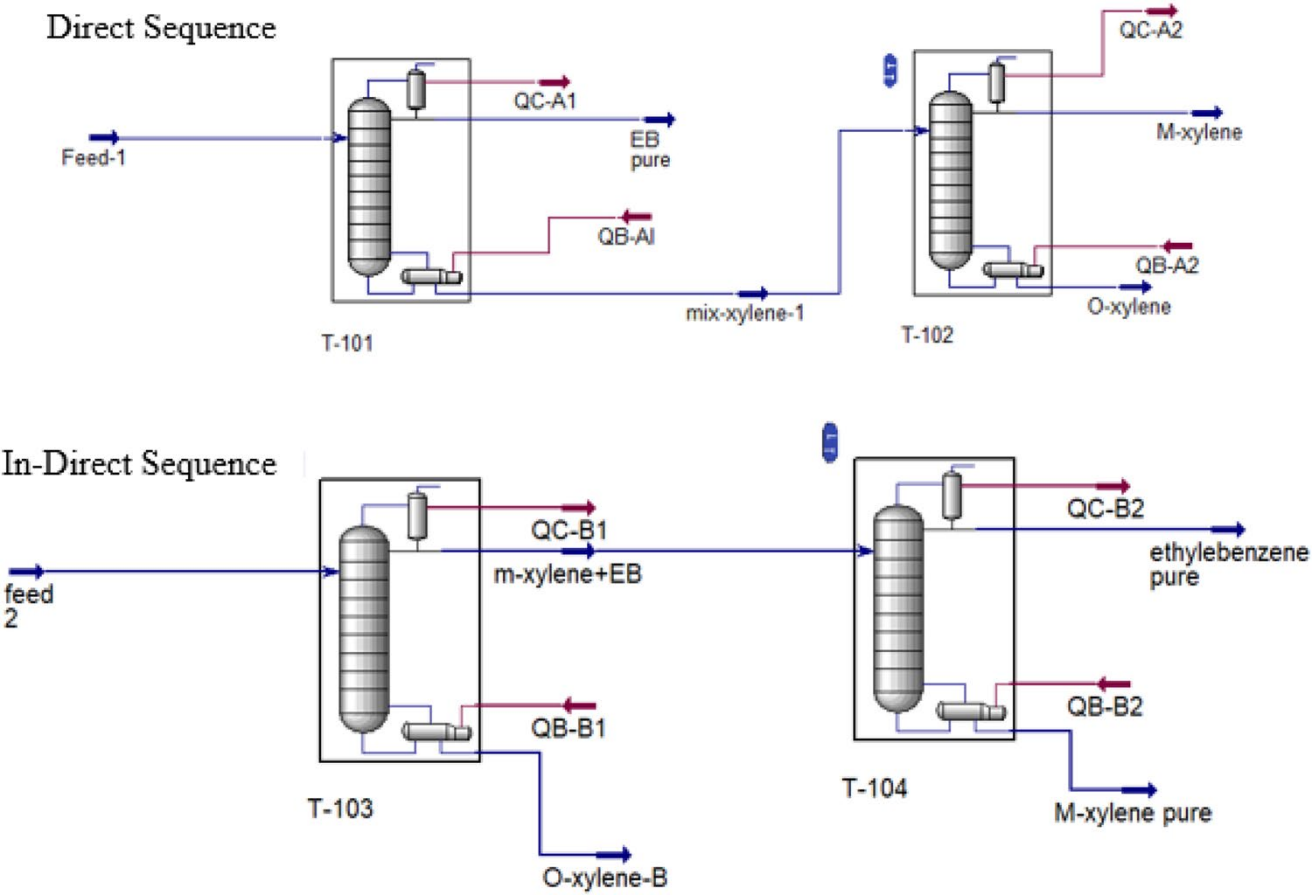
Direct and In-Direct sequence.

**Figure 7:**
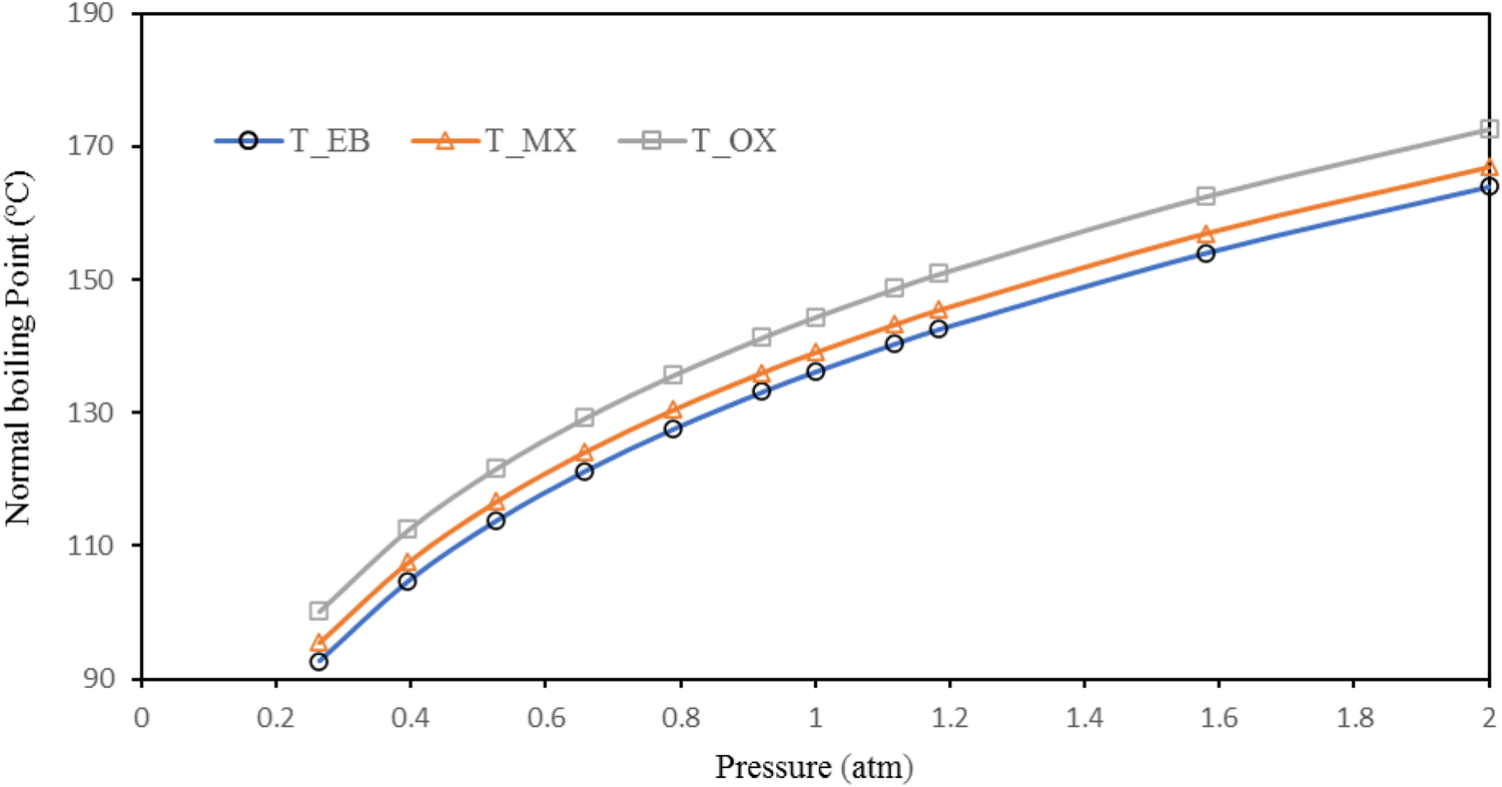
Variation of the MX, OX, and EB boiling points with pressure.

Separation of three-component mixture using distillation, if the boiling points of the components are close to each other, can be done by two direct sequence and in-direct sequence methods (Fig. 6). In this study, both methods have been investigated and the results have been compared from the total number of trays, the condenser and reboiler duties, and also the specifications of the towers, including the diameter and height of the towers, points of view. As can be seen from Table 6, meta-xylene has the highest amount in the feed stream and its boiling point is between the two compounds of ethylbenzene and ortho-xylene. The effect of pressure on the boiling point of these compounds is shown in Fig. 7. As can be seen, with the increase in pressure from 0.25 atm vacuum to the atmospheric pressure range, the boiling point difference of the components increases little with the increase in pressure, but with further increase, the boiling point difference of the components is approximately constant. Accordingly, in this study, atmospheric pressure has been used as the operating pressure. In this study, in the first step, the design of distillation towers was investigated by both methods to achieve 99% purity of meta-xylene.

##### Direct sequencing separation process

The simulation results for the two towers required for this method are summarized in Table 8. In this case, the volatile component is separated from the top of the tower and the heavy components are taken from the bottom of the first tower and enter the second one. In the first distillation tower, 330 trays with a diameter of about 12 m are needed due to the difference in the boiling point of 3℃ between ethylbenzene and meta-xylene. Also, the reboiler and condenser duties of this tower are huge. For the second distillation tower, 125 trays and a reflux ratio of 12 are needed to reach 99% purity of meta-xylene. The lower number of trays is due to the difference of 5℃ in the boiling point of meta and ortho-xylene. In this case, the purity of meta-xylene product from the top of the second distillation tower will reach 99% and the purity of ortho-xylene separated from the bottom of the tower will reach 99.5%. Also, 0.2% of the separated ethylbenzene is coming from the first tower and withdraw along with the meta-xylene flow from the second tower.

**Table 8:** Results of design distillation towers.

	Minimum purity 99% of MX				Minimum purity 96% of MX approach			
	T-101	T-102	T-103	T-104	T-101	T-102	T-103	T-104
No. of Tray	330	125	128	285	190	125	128	185
Feed Flow (m3/h)	50	50	50	50	50	50	50	50
Feed Tray	277	68	50	68	160	68	50	93
Tray Type	Sieve	Sieve	Sieve	Sieve	Sieve	Sieve	Sieve	Sieve
Tray Spacing (m)	0.6096	0.6096	0.6096	0.6096	0.6096	0.6096	0.6096	0.6096
Tower Diameter (m)	12.82	7.201	7.413	6.905	10.14	7.05	6.619	6.282
Condenser Duty (kJ/h)	576,142,141	158,801,476	185,986,026	165,958,629	392,960,574	153,040,056	137,907,161	121,023,928
Reboiler Duty (kJ/h)	585,465,629	158,269,353	194,078,405	166,493,557	376,879,751	151,801,071	120,745,436	121,570,961
Condenser Pressure (bar)	100	100	100	100	100	100	100	100
Reboiler Pressure (bar)	140	120	120	120	140	120	120	120
Condenser Temperature ($^\circ$C)	135.8	138.9	138.6	135.7	135.9	138.9	138.6	145.7
Reboiler Temperature ($^\circ$C)	152.2	150.3	150.3	145.7	152.0	150.3	150.3	135.7
EB-Purity (%)	99	–	–	99	95	–	–	96
EB-Recovery (%)	–	–	–	–	94	–	–	66
MX-Purity (%)	–	98.92	–	99.1	–	98.33	–	96
MX-Recovery (%)	–	–	–	–	–	99	–	99
OX-Purity (%)	–	99	99	–	–	99	98	–
OX-Recovery (%)	–	–	–	–	–	90.6	90.5	–

##### In-direct sequencing separation process

The results obtained from the design of distillation towers by in-direct method are shown in Table 8. In this case, in the first tower, ortho-xylene is removed from the bottom of the tower, and ethylbenzene and meta-xylene will be separated from the top of the first tower. Then, the stream containing ethylbenzene and meta-xylene is entered into the second tower for further separation, and finally meta-xylene is obtained from the bottom of the second tower. It can also be seen here that in order to achieve a purity of 99% meta-xylene, the second distillation tower should have 290 trays. Also, the reboiler and condenser duties are high.

According to the results obtained above, the distillation method cannot be operationally applicable to achieve the purity of 99% of meta-xylene. For this reason, in order to make the process operational, in the next step, the meta-xylene product with the purity of 96% was considered. Reducing the purity of the final product, by reducing the number of trays and the diameter of the distillation tower, are advanced the process towards operationalization.

##### Separation based on 96% of meta-xylene product (as minimum purity)

Table 8 shows the results obtained to reach 96% pure meta-xylene by both direct and in-direct methods. In this part, the number of trays needed for the separation tower of meta-xylene from ethylbenzene or meta-xylene from ortho-xylene are decreased. Also, the amount of reflux required and the duties required for the condenser and reboiler are reduced.

As can be seen from Table 8, the purity of meta-xylene in the direct sequence method is higher than the in-direct sequence method. On the other hand, the recovery of ethylbenzene product in the in-direct sequence mode is much less than the direct sequence mode. The reason for this phenomenon can also be related to the close boiling points of ethylbenzene and meta-xylene. Therefore, in the case of direct sequence where ethylbenzene is first separated from meta and ortho-xylene in the first tower, in the second tower it is relatively easier to separate two xylene compounds with large differences between their boiling points. Therefore, it can be seen that in the case of direct sequence, three products are separated from each other with a higher recovery.

### Economic evaluation of the process

After obtaining the specifications of the process to achieve 96% purity of meta-xylene using both direct and in-direct methods, the economic analysis was used to find the optimal and feasible process. Aspen Process Economic Analyzer was utilized for this aim^33–36^.

The Table 9 shows the prices of each equipment needed in distillation towers according to the capacity and specification obtained for both methods. As can be seen from Table 9, the cost of purchasing distillation towers in direct sequence method is twice in-direct sequence process, which the main reason is high diameter of the tower (ca. 10 m) to separate EB from MX and OX. The cost of purchasing equipment is based on carbon steel material, and the cost of reboiler and condenser equipment are calculated based on their duties^33–36^.

**Table 9:** Equipment prices^33–36^.

In-direct sequence			Direct dequence		
No	Equipment	Equipment Cost [USD]	No	Equipment	Equipment Cost [USD]
1	Main Tower_@T-103	3,965,800	1	Reboiler_@T-101-reb	3,589,300
2	Condenser_@T-103-cond	109,600	2	Condenser_@T-101-cond	298,900
3	Condenser_@T-103-cond acc	78,700	3	Condenser_@T-101-cond acc	144,300
4	Condenser_@T-103-reflux pump	43,400	4	Condenser_@T-101-reflux pump	127,500
5	Reboiler_@T-103-reb	1,018,200	5	Main Tower_@T-101	12,390,900
6	Reboiler_@T-104-reb	777,200	6	Reboiler_@T-102-reb	1,294,100
7	Condenser_@T-104-cond	103,500	7	Main Tower_@T-102	4,192,200
8	Condenser_@T-104-cond acc	74,500	8	Condenser_@T-102-cond	117,300
9	Condenser_@T-104-reflux pump	32,500	9	Condenser_@T-102-cond acc	79,500
10	Main Tower_@T-104	6,526,700	10	Condenser_@T-102-reflux pump	44,900
Total purchased costs		12,730,100	Total purchased costs		22,278,900

Also, fixed costs estimated and gathered from the references can be seen in Table 10. It can be obvious, the fixed costs for the separation using the in-direct sequence method are much lower than the separation costs using the direct sequence method. From the total investment cost, which is the sum of fixed costs (cell A) and working capital (cell B), it can be seen that the difference in the total cost between the two separation methods is more than 52 million dollars. Furthermore, according to the reported results, the amount of profit of each method has been investigated according to this difference in investment cost.

**Table 10:** Fixed Cost estimation.

Investments factors			Direct seq. ($)	In-direct seq. ($)
C1	Total mani equipment cost	Purchased Cost	22,278,900	12,730,100
C2	Total main + Auxiliary Equipment cost	(1.1 * C1)	24,506,790	14,003,110
A	Total Fixed Capital Investment	A1 + A2	106,359,469	**60,773,497**
A1	Total Direct plant cost	1 to 9	75,480,913	43,129,579
1	Delivered main equipment (includes auxiliary equipment)	100%	24,506,790	14,003,110
2	Purchased-equipment installation	39%	9,557,648	5,461,213
3	Instrumentation and controls (installed)	26%	6,371,765	3,640,809
4	Piping (installed)	31%	7,597,105	4,340,964
5	Electrical (installed)	10%	2,450,679	1,400,311
6	Buildings (including services)	29%	7,106,969	4,060,902
7	Yard improvements	12%	2,940,815	1,680,373
8	Service facilities (installed)	55%	13,478,735	7,701,711
9	land	6%	1,470,407	840,187
A2	Total Indirect plant cost	10 to 14	30,878,555	17,643,919
10	Engineering and supervision	32%	7,842,173	4,480,995
11	Construction expenses	34%	8,332,309	4,761,057
12	Legal expenses	4%	980,272	560,124
13	Contractor’s fee	19%	4,656,290	2,660,591
14	Contingency	37%	9,067,512	5,181,151
B	Working Capital	15 + 16	21,271,894	**12,154,699**
15	About 15% of total fixed capital investment	75%	15,953,920	9,116,025
16	Safety and hazard analyses	5%	5,317,973	3,038,675
	TOTAL CAPITAL INVESTMENT	A + B	127,631,362	72,928,197

To determine the production costs, the price of feed and products in the average world price market has been used as the basis (Table 11). By means of production costs and required fixed costs, the amount of product production and profit from sales based on the simulated processes in direct and in-direct sequence methods is calculated, which are shown in Table 12 and 13, respectively.

**Table 11:** Average world price xylene price.

Material	Cost ($/kg)
Raffinate Feed	0.7000
Meta-xylene	0.9700
Orto-Xylene	0.8793
Ethylbenzene	0.8952

**Table 12:** Product production costs.

Total annual product cost for the process		value	Direct Seq. ($)	In-Direct Seq. ($)
C	MANUFACTURING COST	C1 + C2 + C3	344,499,790	255,526,515
C1	DIRECT PRODUCTION COSTS	1 to 8	328,479,881	246,344,503
1	Raw materials		278,050,752	218,937,600
2	Operating labor		93,600	93,600
3	Direct supervisory and clerical labor	17.5% of item 2	16,380	16,380
4	Utilities (calculated)		33,390,299	15,361,961
5	Maintenance and repairs	3.5% of fixed-capital investment	3,722,581	2,127,072
6	Operating supplies	15% of item 5	558,387	319,061
7	Laboratory charges	15% of item 2	14,040	14,040
8	Patents and royalties	4% of item 1 to 7	12,633,842	9,474,789
C2	INDIRECT PRODUCTION COSTS	9 to 11	13,720,371	7,839,781
9	Depreciation	10% of fixed-capital investment	10,635,947	6,077,350
10	Local taxes	2.5% of fixed-capital investment	2,658,987	1,519,337
11	Insurance	0.4% of fixed-capital investment	425,438	243,094
C3	PLANT-OVERHEAD COSTS	60% of (item 2 + 3 + 5)	2,299,537	1,342,231
D	GENERAL EXPENSES	14 to 16	27,292,495	27,167,595
14	Administrative costs	15% of (item 2 + 3 + 5)	574,884	335,558
15	Distribution and selling costs	3% of sales	10,019,104	10,062,014
16	Research and development costs	5% of sales	16,698,507	16,770,023
	Total Production Cost	C + D	371,792,285	282,694,110

**Table 13:** Profit of the sale production.

	In-Direct Seq			Direct Seq		
	EB (ton/hr)	MX (ton/hr)	OX (ton/hr)	EB (ton/hr)	MX (ton/hr)	OX (ton/hr)
Product	2.36	37.83	3.25	3.33	36.86	3.25
Price ($/unit)	895.2	970	879.3	895.2	970	879.3
Total price ($/hr)	2112.672	36,695.1	2857.725	2981.016	35,754.2	2857.725

By determining investment costs, production costs, income from the sales, the economic parameters of these processes were calculated (Table 14). According to the obtained results, it was determined that in the in-direct sequence method, despite the fact that the purity of the MX product is lower, the rate of return on investment and IRR is higher, while the period of return on investment is lower.

**Table 14:** Economic parameters of the process.

	Direct Seq	In-Direct Seq
Economic index	$	$
Total utility costs ($/year)	33,390,299	15,361,961
Total capital investment costs ($)	127,631,362	72,928,197
Sale costs ($/year)	299,469,175	299,991,578
Raw material costs ($/year)	218,937,600	218,937,600
plant Life (year)	20	20
Total production cost ($/year)	307,554,530	279,861,399
Annual profit ($/year)	−8,085,355	20,130,179
Internal rate of retun (%)	–	27.4
P.O.P (year)	–	4.0

## Conclusion

In this research, the possibility of revamping the ELUXYL para-xylene separation plants using Ba-exchanged faujasite adsorbent to separate meta-xylene with the help of changing the operating conditions (temperature and pressure) was investigated. In the situation where the feed is mixed-xylene, the adsorbent used in existing plant is suitable for separating para-xylene, and it cannot be able to change its selectivity towards meta-xylene under any operating conditions of pressure and temperature. Also, in the absence of para-xylene in the feed (Raffinate feed) at the temperature and pressure of 185℃ and 8.7 bar, respectively, selectivity of the adsorbent is in a favor of meta-xylene. The selectivity factor for meta-xylene to ethylbenzene and ortho-xylene were 1.15 and 1.8, respectively. Using the design and simulation of the unit utilizing the obtained experimental data, it was found that the selectivity of meta-xylene is not suitable enough for this process. It can only slightly improve the purity of meta-xylene from 60 to 83%. Finally, it was found that by integrating the distillation method with the revamped plant through the in-direct sequencing method, purity of the meta-xylene can be increased from 83 to 96% with the suitable economical results.

## Supplementary Information

Supplementary Information.

## Data Availability

Authors can confirm that all relevant data are included in this article and its Supplementary Information file.

## Abbreviations

PX: Para-xylene
MX: Meta-xylene
OX: Ortho-xylene
EB: Ethylbenzene
PDEB: Para-diethyl benzene
SMB: Simulated moving bed
IPA: Iso phthalic acid
PET: Poly ethylene terephthalate
DOE: Design of experiment
RSM: Response surface method
