# Supplementary material for: Experimental and techno-economic investigation of industrial para-xylene plant revamping to produce meta-xylene
Source: Sci Rep. 2023 Aug 2;13:12534. doi: 10.1038/s41598-023-39526-3 (PMC10397266; doi:10.1038/s41598-023-39526-3)
Supplement: Supplementary file 1 — Supplementary Information. [file 41598_2023_39526_MOESM1_ESM.docx]

**Experimental and techno-economic investigation of industrial para-xylene plant revamping to produce meta-xylene**

MohammadReza Khosravi-Nikou^a,^*, Ali Shahmoradi^a^, Ahmad Shariati^a^, Meysam Hajilari^a^, Mahsa Malek-Mahmoudi^a^, Nemat-Allah Jafari^b^, Abdollah Sheikh-Nezhad^b^

^a^ Department of Gas Engineering, Ahwaz Faculty of Petroleum, Petroleum University of Technology, Ahwaz, Iran

^b^ Shahid Tondgouyan Petrochemical Company, Mahshahr, Iran

* Corresponding author

E-mail address: [mr.khosravi@put.ac.ir](mailto:mr.khosravi@put.ac.ir)

[khosravi_m@yahoo.com](mailto:khosravi_m@yahoo.com)

1. Batch and dynamic set-ups
   1. Set-ups

The schemes of batch and dynamic set-ups are proposed in figure S-1 and figure S-2, respectively. In the batch set-up, controlling the temperature of the chamber is done by heating element and PID controller. For the aim of full contact between feed and adsorbent, an impeller was utilized. After introducing feed and adsorbent in chamber, argon gas was used for pressurizing as an inert medium. During each experiment, pressure and temperature were adjusted to ensure that mixture is in the form of liquid. Sampling system comprised of two valves and a line linked to vessel. Cooling water (ice) bath were utilized to reducing sample temperature to ambient before introducing to the GC for analyzing.

The dynamic adsorption set-up comprised of fixed-bed column, thermal jacket (for the aim of temperature controlling), connection lines and valves were used for determination of breakthrough analysis. Configuration of the set-up is presented in table S-1. Para-diethylbenzene (desorbent) saturate column in the first step, then by changing the position of the valve 1, xylene feed goes through column. Sample collecting is just started by introducing xylene feed to the column. The samples were collected during 2 hours with a sampling of 30 s.


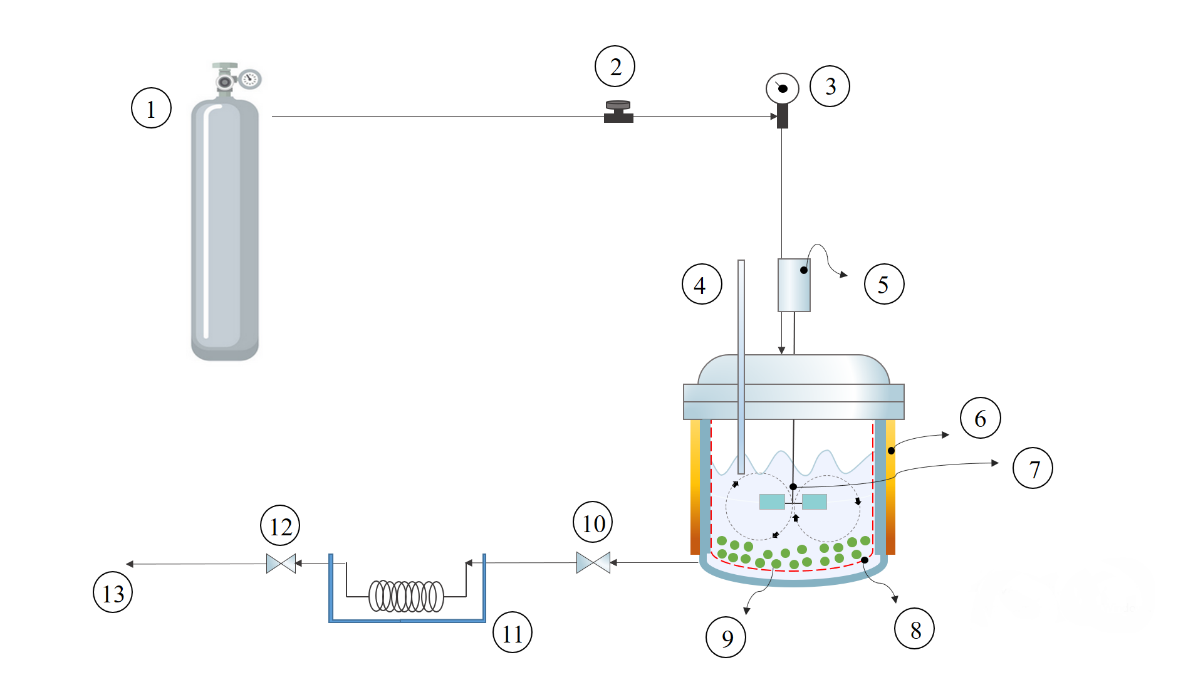


Figure S-1. Schematic of batch setup. (1) Inert gas (argon), (2) needle valve, (3) pressure gauge (4) thermometer, (5) motor, (6) heat element, (7) impeller, (8) baffle, (9) adsorbent, (10) needle valve, (11) water bath, (12) needle valve, (13) sampling.


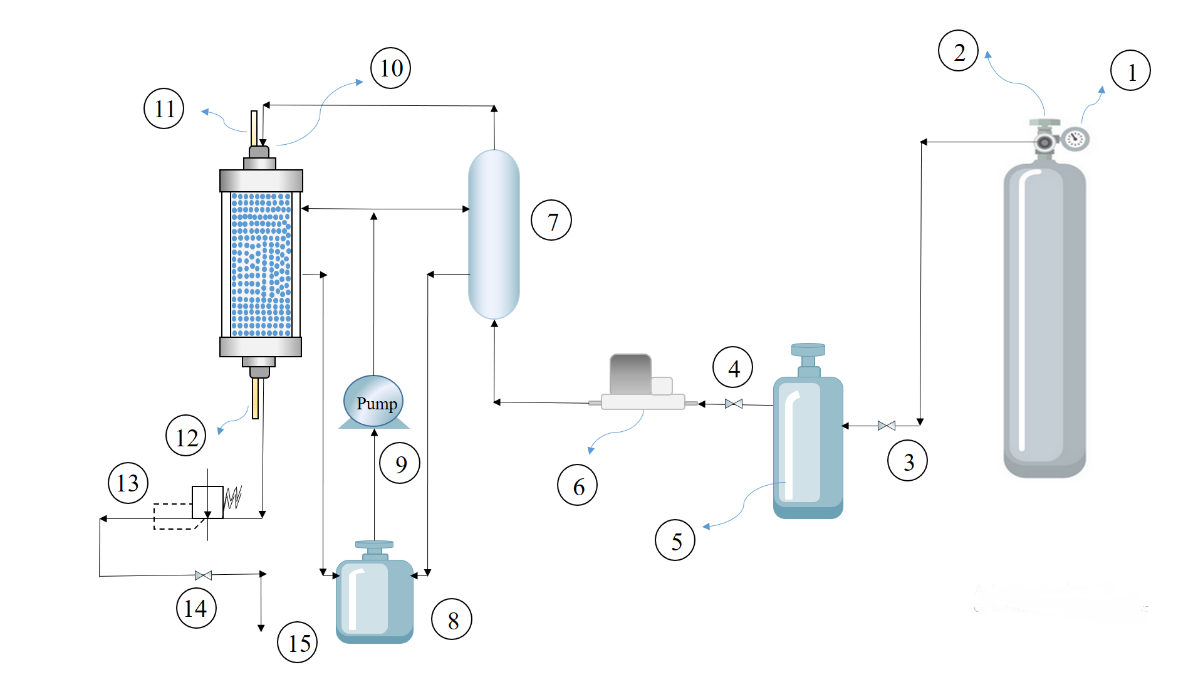


Figure S-2. Schematic of dynamic setup. (1) inert gas (argon), (2) gauge, (3) needle valve (4) needle valve, (5) feed solution, (6) MFC, (7) heating column, (8) oil tank, (9) pump, (10) adsorption column (11) thermometer, (12) thermometer, (13) pressure reducer, (14) needle valve, (15) sampling.

Table S-1. Characteristics of the packed column.

| Length (cm) | 30 |
| --- | --- |
| Diameter (cm) | 1.5 |
| Volume (cm^3^) | 70.65 |
| Mass of adsorbent (gr) | 30.9 |
| Adsorption particle diameter (mm) | 0.47 |

### Method of analysis

Compositions concentration were measured at the input and output of the set-ups by means of YL6100 GC Gas Chromatography. It is equipped with a capillary column (Supra Wax 280 with 60 m length, 0.32 mm internal diameter, and 1 μm film thickness) and an ionized flame detector (FID), having helium as a carrier gas. 1 μl of the output sample was injected into the column of the device, the temperature rising from 60 to 220 at a rate of 5c/min. The injection point temperature is 210, and the temperature of detector FID is 230. All weights were measured using a scale with an error of ± 0.0001. The mass-based adsorption loadings are calculated as follows:

$q_{i}=\frac{m_{sol}\left( C_{O}-C_{e} \right)i}{m_{ads}}$ (1)

Where $q_{i}$is loading of adsorption (mg_adsorbed_/g_adsorbent_), $C_{O}$ is initial concentration (wt%) of component *i,* $C_{e}$ equilibrium concentration (wt%) of component *i*, $m_{sol}$ is initial concentration of feed (gr), and $m_{ads}$is mass of adsorbent. The adsorbed concentration calculated by Eq.1 includes the solute in the pores. However, due to the fact that the amount of adsorbate molecules in the pores compared to the amount of material introduced to batch set-up in each experiment is small (about less than 2 %), so it does not have a significant effect on the amount reported for the adsorbed amount and can be neglected[1].

1. Modeling of simulated moving bed

For the aim of designing SMB process, due to the existence of mass transfer resistance, triangle theory was utilized to reach initial guess[2, 3]. For this purpose, ASPEN Chromatography was used. In the case of triangle theory, flowrates are computing by tuning zone 2 and 3 flowrates based on TMB approach modeling. TMB approach modeling considers bellow equations[3, 4]:

Mass balance in bulk liquid phase for the component *i*:

$\frac{\partial c_{ij}}{\partial t}+(\frac{1-\varepsilon_{i}}{\varepsilon_{i}})\frac{\partial q_{ij}}{\partial t}=D_{{ax}_{j}}\frac{\partial^{2}c_{ij}}{\partial x^{2}}-\frac{\partial(v_{j} c_{ij})}{\partial x}-u_{s}(\frac{1-\varepsilon_{i}}{\varepsilon_{i}})\frac{\partial q_{ij}}{\partial x}$ (1)

Mass balance in the adsorbent particle for the component *i*:

$\frac{\partial q_{ij}}{\partial t}= u_{s}\frac{\partial q_{ij}}{\partial x}+ k_{l,i}(q_{ij}^{*}-q_{ij})$ (2)

The initial and boundary conditions defined as:

$t=0 : c_{ij}=q_{ij}=0$ (3)

$x=0 : v_{j}c_{ij}- D_{{ax}_{j}}\frac{\partial c_{ij}}{\partial x}= v_{j}c_{ij}^{in}$ (4)

$x=l : \frac{\partial c_{ij}}{\partial x}=0$ (5)

The adsorption equilibrium is described with extended Longmuir isotherm:

$q_{i,k}=\frac{q_{m_{i}}*K_{i}{*c}_{p_{i,k}}}{1+\sum_{1}^{n} K_{i}{*c}_{p_{i,k}}}$ (6)

Where $c_{i,j}$are the liquid phase and average pore (liquid adsorbed) concentration of component *i* in the section *j*.$q_{i,j}$ and $q_{ij}^{*}$ are the average and equilibrium adsorbed concentration of component *i* in the *j* column where $q_{m_{i}} and K_{i}$ are saturation capacity and constant parameter of Langmuir adsorption isotherm for component *i* in the *j* section. $\varepsilon_{i}$*,* $\varepsilon_{p}$*,* and $\rho_{p}$ are the bed porosity, particle porosity, and density of the particle, respectively. $v_{j}$ and $u_{s}$ are the fluid and solid (adsorbent bed) velocities of *j* section. $D_{ax,j} and k_{l,i}$ are axial dispersion coefficient and global mass transfer coefficient for *ith* component, respectively.

By implementing data driven for model from experiments (Table 4) and configuration of the SMB (Table 2), separation region which is shown in figure S-3, is obtained.


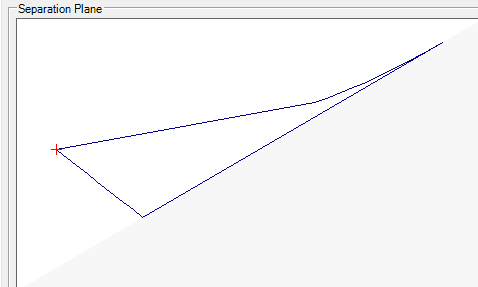


Figure S-3. Separation region obtained from triangle theory, by the help of ASPEN Chromatography software.

After obtaining initial values for flowrates, optimum condition can be accessed through dynamic optimization. For this aim, SMB approach modeling was implemented through ASPEN Chromatography to find optimum condition regarding high performance parameters. The detail of the SMB approach modeling equations are presented below[4-6]:

Mass balance in bulk liquid phase for the component *i*:

$\frac{\partial c_{i,k}}{\partial t}+\frac{1-\varepsilon_{i}}{\varepsilon_{i}}k_{l,i}(c_{i,k}-c_{p_{i,k}})+\frac{\partial(v_{k}^{⃰}\times c_{i,k})}{\partial z}-D_{L_{k}}\frac{\partial^{2}c_{i,k}}{\partial z^{2}}=0$ (7)

Mass balance in the adsorbent particle for the component *i*:

$k_{l,i}(c_{i,k}-c_{p_{i,k}})=\varepsilon_{p}\frac{\partial c_{p_{i,k}}}{\partial t}+\rho_{p}\frac{\partial q_{i,k}}{\partial t}$ (8)

The initial and boundary conditions defined as:

$t = 0: c_{i,k}=c_{p_{i,k}}= 0 and q_{i,k}=0$ (9)

$z=0: v_{k}^{⃰}c_{i,k}- D_{L_{k}}\frac{\partial c_{i,k}}{\partial z}|_{z=0}= v_{k}^{⃰}C_{i,k}^{in}$ (10)

$z=L_{j}: \frac{{\partial C}_{i,k}}{\partial z}|_{z=L_{k}}=0$ (11)

Where $c_{i,k} and c_{p_{i,k}}$ are the liquid phase and average pore (liquid adsorbed) concentration of component *i* in the *kth* column.$q_{i,k}$ is the average adsorbed concentration of component *i* in the *kth*. $v_{k}^{⃰}$ is the interstitial velocity of liquid in the *kth* column. $D_{L_{k}} and k_{l,i}$ are axial dispersion coefficient and global mass transfer coefficient for *ith* component, respectively.

1. Experimental Design

Table S-2. experimental design’s matrix and the experimental values of the responses

| Std | Run | Block | Factor 1 | Factor 2 | Response 1 | Response 2 | Response 3 |
| --- | --- | --- | --- | --- | --- | --- | --- |
|  |  |  | A:Temperature | B:Pressure | EB | MX | OX |
|  |  |  | C | Bar | mg/g | mg/g | mg/g |
| 6 | 1 | Block 1 | 200 | 10 | 12.878 | 63.502 | 19.126 |
| 2 | 2 | Block 1 | 185 | 8 | 11.304 | 57.836 | 17.392 |
| 9 | 3 | Block 1 | 137.5 | 10 | 9.06 | 35.422 | 11.048 |
| 4 | 4 | Block 1 | 200 | 12 | 12.6927 | 52.5027 | 21.4147 |
| 1 | 5 | Block 1 | 75 | 8 | 20.172 | 75.34 | 35.2 |
| 8 | 6 | Block 1 | 137.5 | 12 | 7.592 | 29.824 | 8.106 |
| 7 | 7 | Block 1 | 137.5 | 8 | 10.692 | 59.784 | 19.09 |
| 3 | 8 | Block 1 | 75 | 12 | 13.884 | 62.344 | 18.714 |
| 5 | 9 | Block 1 | 75 | 10 | 15.488 | 74.052 | 22.492 |

After introducing data to the “design Expert” software, data analysis were implemented. Results are presented in table S-3, S-4, and S-5.

Table S-3. Data analysis for EB response

| Sequential Model Sum of Squares [Type I] | | | | | | |
| --- | --- | --- | --- | --- | --- | --- |
|  | Sum of |  | Mean | F | p-value |  |
| Source | Squares | df | Square | Value | Prob > F |  |
| Mean vs Total | 1437.994 | 1 | 1437.994 |  |  |  |
| Linear vs Mean | 36.68047 | 2 | 18.34024 | 1.494176 | 0.2974 |  |
| 2FI vs Linear | 18.07372 | 1 | 18.07372 | 1.626119 | 0.2583 |  |
| Quadratic vs 2FI | 54.48371 | 2 | 27.24186 | 75.0135 | 0.0027 | Suggested |
| Cubic vs Quadratic | 1.089478 | 3 | 0.363159 |  |  | Aliased |
| Residual | 0 | 0 |  |  |  |  |
| Total | 1548.321 | 9 | 172.0357 |  |  |  |

Table S-4. Data analysis for MX response

| Sequential Model Sum of Squares [Type I] | | | | | | |
| --- | --- | --- | --- | --- | --- | --- |
|  | Sum of |  | Mean | F | p-value |  |
| Source | Squares | df | Square | Value | Prob > F |  |
| Mean vs Total | 28968.8 | 1 | 28968.8 |  |  |  |
| Linear vs Mean | 624.6336 | 2 | 312.3168 | 1.435377 | 0.3094 |  |
| 2FI vs Linear | 22.21277 | 1 | 22.21277 | 0.086546 | 0.7804 |  |
| Quadratic vs 2FI | 1111.755 | 2 | 555.8777 | 9.721365 | 0.0489 | Suggested |
| Cubic vs Quadratic | 171.5431 | 3 | 57.18103 |  |  | Aliased |
| Residual | 0 | 0 |  |  |  |  |
| Total | 30898.94 | 9 | 3433.216 |  |  |  |

Table S-5. Data analysis for OX response

| Sequential Model Sum of Squares [Type I] | | | | | | |
| --- | --- | --- | --- | --- | --- | --- |
|  | Sum of |  | Mean | F | p-value |  |
| Source | Squares | df | Square | Value | Prob > F |  |
| Mean vs Total | 3309.42 | 1 | 3309.42 |  |  |  |
| Linear vs Mean | 144.2511 | 2 | 72.12556 | 1.350075 | 0.3280 |  |
| 2FI vs Linear | 119.048 | 1 | 119.048 | 2.95416 | 0.1463 |  |
| Quadratic vs 2FI | 194.675 | 2 | 97.33752 | 42.83505 | 0.0062 | Suggested |
| Cubic vs Quadratic | 6.817141 | 3 | 2.27238 |  |  | Aliased |
| Residual | 0 | 0 |  |  |  |  |
| Total | 3774.211 | 9 | 419.3568 |  |  |  |

As can be seen from resulted data, quadratic model can describe experimental values of the responses more reasonable. By choosing this model, analysis of variance (ANOVA) for three responses were implemented. The results of this analysis are presented in table S-6 to table S-11.

Table S-6. EB ANOVA results

| ANOVA for Response Surface Quadratic Model | | | | | | |
| --- | --- | --- | --- | --- | --- | --- |
| Analysis of variance table [Partial sum of squares - Type III] | | | | | | |
|  | Sum of |  | Mean | F | p-value |  |
| Source | Squares | df | Square | Value | Prob > F |  |
| Model | 109.2379 | 5 | 21.84758 | 60.15976 | 0.0033 | significant |
| A-Temp | 18.24687 | 1 | 18.24687 | 50.24481 | 0.0058 |  |
| B-Pressure | 14.82497 | 1 | 14.82497 | 40.82221 | 0.0078 |  |
| AB | 7.826345 | 1 | 7.826345 | 21.55072 | 0.0188 |  |
| A^2 | 54.4757 | 1 | 54.4757 | 150.0049 | 0.0012 |  |
| B^2 | 0.509289 | 1 | 0.509289 | 1.402385 | 0.3216 |  |
| Residual | 1.089478 | 3 | 0.363159 |  |  |  |
| Cor Total | 110.3274 | 8 |  |  |  |  |

Table S-7. Statistical parameters calculated from quadratic model for EB

| Std. Dev. | 0.602627 | R-Squared | 0.990125 |
| --- | --- | --- | --- |
| Mean | 12.6403 | Adj R-Squared | 0.973667 |
| C.V. % | 4.767507 | Pred R-Squared | 0.879818 |
| PRESS | 13.25941 | Adeq Precision | 24.6719 |

Table S-8. MX ANOVA results

| ANOVA for Response Surface Reduced Quadratic Model | | | | | | |
| --- | --- | --- | --- | --- | --- | --- |
| Analysis of variance table [Partial sum of squares - Type III] | | | | | | |
|  | Sum of |  | Mean | F | p-value |  |
| Source | Squares | df | Square | Value | Prob > F |  |
| Model | 1758.488 | 4 | 439.622 | 10.24422 | 0.0223 | significant |
| A-Temp | 109.3053 | 1 | 109.3053 | 2.547069 | 0.1857 |  |
| B-Pressure | 546.9261 | 1 | 546.9261 | 12.74465 | 0.0234 |  |
| AB | 2.159403 | 1 | 2.159403 | 0.050319 | 0.8335 |  |
| A^2 | 1111.642 | 1 | 1111.642 | 25.90384 | 0.0070 |  |
| Residual | 171.6567 | 4 | 42.91417 |  |  |  |
| Cor Total | 1930.145 | 8 |  |  |  |  |

Table S-9. Statistical parameters calculated from quadratic model for MX

| Std. Dev. | 6.550891 | R-Squared | 0.911065 |
| --- | --- | --- | --- |
| Mean | 56.73407 | Adj R-Squared | 0.822131 |
| C.V. % | 11.54666 | Pred R-Squared | 0.539498 |
| PRESS | 888.836 | Adeq Precision | 9.878972 |

Table S-10. OX ANOVA results

| ANOVA for Response Surface Quadratic Model | | | | | | |
| --- | --- | --- | --- | --- | --- | --- |
| Analysis of variance table [Partial sum of squares - Type III] | | | | | | |
|  | Sum of |  | Mean | F | p-value |  |
| Source | Squares | df | Square | Value | Prob > F |  |
| Model | 457.9742 | 5 | 91.59484 | 40.30788 | 0.0060 | significant |
| A-Temp | 35.79336 | 1 | 35.79336 | 15.75148 | 0.0286 |  |
| B-Pressure | 110.9757 | 1 | 110.9757 | 48.83676 | 0.0060 |  |
| AB | 65.29581 | 1 | 65.29581 | 28.73454 | 0.0127 |  |
| A^2 | 186.1985 | 1 | 186.1985 | 81.93983 | 0.0028 |  |
| B^2 | 16.447 | 1 | 16.447 | 7.237783 | 0.0744 |  |
| Residual | 6.817141 | 3 | 2.27238 |  |  |  |
| Cor Total | 464.7914 | 8 |  |  |  |  |

Table S-11. Statistical parameters calculated from quadratic model for OX

| Std. Dev. | 1.507442 | R-Squared | 0.985333 |
| --- | --- | --- | --- |
| Mean | 19.17585 | Adj R-Squared | 0.960888 |
| C.V. % | 7.861146 | Pred R-Squared | 0.833082 |
| PRESS | 77.58219 | Adeq Precision | 21.14074 |

As can be seen from above results, all three surfaces are acceptable by considering P < 0.05. this shows that quadratic model is good model for describing experimental adsorption behavior of MX, OX, and EB.

References

[1] M. S. Silva, J. P. Mota, and A. E. Rodrigues, "Adsorption equilibrium and kinetics of the Parex'feed and desorbent streams from batch experiments," *Chemical Engineering & Technology,* vol. 37, no. 9, pp. 1541-1551, 2014.

[2] M. Minceva and A. E. Rodrigues, "Influence of the transfer line dead volume on the performance of an industrial scale simulated moving bed for p-xylene separation," *Separation science and technology,* vol. 38, no. 7, pp. 1463-1497, 2003.

[3] M. Minceva and A. E. Rodrigues, "Modeling and simulation of a simulated moving bed for the separation of p-xylene," *Industrial & engineering chemistry research,* vol. 41, no. 14, pp. 3454-3461, 2002.

[4] A. Rodrigues, *Simulated moving bed technology: principles, design and process applications*. Butterworth-Heinemann, 2015.

[5] A. Shahmoradi, M. R. Khosravi-Nikou, M. Aghajani, A. Shariati, and S. Saadi, "Mathematical modeling and optimization of industrial scale ELUXYL simulated moving bed (SMB)," *Separation and Purification Technology,* vol. 248, p. 116961, 2020.

[6] Y. Shen, Q. Fu, D. Zhang, and P. Na, "A systematic simulation and optimization of an industrial-scale p-xylene simulated moving bed process," *Separation and Purification Technology,* vol. 191, pp. 48-60, 2018.
